# The validity and reliability of quadriceps twitch force as a measure of skeletal muscle fatigue while cycling

**DOI:** 10.1002/ejsc.12181

**Published:** 2024-08-08

**Authors:** Keenan B. MacDougall, Saied J. Aboodarda, Paulina H. Westergard, Brian R. MacIntosh

**Affiliations:** ^1^ Faculty of Kinesiology University of Calgary Calgary Alberta Canada

**Keywords:** cycling exercise, dynamic muscle function, muscle contractile function, nerve stimulation, skeletal muscle fatigue

## Abstract

The measurement of skeletal muscle fatigue in response to cycling exercise is commonly done in isometric conditions, potentially limiting its ecological validity, and creating challenges in monitoring the time course of muscle fatigue across an exercise bout. This study aimed to determine if muscle fatigue could be reliably assessed by measuring quadriceps twitch force evoked while pedaling, using instrumented pedals. Nine participants completed three laboratory visits: a step incremental test to determine power output at lactate threshold, and on separate occasions, two constant‐intensity bouts at a power output 10% above lactate threshold. Femoral nerve electrical stimulation was applied to elicit quadriceps twitch force both while pedaling (dynamic) and at rest (isometric). The test–retest reliability of the dynamic twitch forces and the agreement between the dynamic and isometric twitch forces were evaluated. Dynamic twitch force was found to have excellent reliability in an unfatigued state (intraclass correlation coefficient (ICC) = 0.920 and mean coefficient of variation (CV) = 7.5%), and maintained good reliability at task failure (ICC = 0.846 and mean CV = 11.5%). When comparing dynamic to isometric twitch forces across the task, there was a greater relative decline in the dynamic condition (*P* = 0.001). However, when data were normalized to the 5 min timepoint when potentiation between conditions was presumed to be more similar, this difference disappeared (*P* = 0.207). The reliability of this method was shown to be commensurate with the gold standard method utilizing seated isometric dynamometers and offers a new avenue to monitor the kinetics of muscle fatigue during cycling in real time.

## Highlights

- The measurement of skeletal muscle fatigue during cycling exercise typically requires stopping the exercise task, potentially confounding the measurement, and limiting the frequency at which measurements can be made
- We have developed a novel method of assessing skeletal muscle fatigue which can be done while allowing cycling exercise to continue uninterrupted
- The reliability of this method was shown to be commensurate with the gold standard method utilizing seated isometric dynamometers

## 1 INTRODUCTION

Skeletal muscle fatigue is typically understood as a reversible decline in muscle contractile function as a result of prior activity, regardless of whether the current task can be sustained (Allen et al., 2008; Bishop, 2012). Within the muscle fibers, fatigue develops because of metabolic changes during periods of high energy demand (Cheng et al., 2018). The high rates of ATP turnover and reliance on anaerobic metabolism can subsequently lead to the accumulation of metabolites, such as inorganic phosphate or hydrogen ions, an accelerated depletion of muscle glycogen as well as the production of reactive oxygen/nitrogen species (Allen et al., 2008; Cheng et al., 2018). These metabolic disturbances may all contribute by various mechanisms to the impairment of muscle contractile function observed during exercise (Allen et al., 2008; Cheng et al., 2018).

The objective measurement of skeletal muscle fatigue during exercise is of interest for many reasons. For example, fatigue may be assessed to determine the impact of various training interventions (Aboodarda et al., 2018; Mira et al., 2018; O’Leary et al., 2017), warm‐up strategies (do Nascimento Salvador et al., 2018), nutritional interventions (Dittrich et al., 2021; Milioni et al., 2019; Schäfer et al., 2019), or environmental conditions (Girard et al., 2013; Mira et al., 2020). Additionally, it may be of interest to study muscle fatigue in various clinical populations (Bachasson, Guinot, et al., 2013; Buller et al., 1991) or to determine the relationship between fatigue development and other physiological measures such as oxygen consumption (Cannon et al., 2011; de Almeida Azevedo et al., 2022; do Nascimento Salvador et al., 2018; Gajanand et al., 2020; Keir et al., 2016). In the majority of the studies exploring skeletal muscle fatigue during cycling exercise, fatigue was measured by moving from a cycle ergometer to a separate seated (isometric) dynamometer, allowing voluntary and electrically evoked muscle force to be measured (Dittrich et al., 2021; Gajanand et al., 2020; Girard et al., 2013; Keir et al., 2016; Milioni et al., 2019; O’Leary et al., 2017; Schäfer et al., 2019). A major concern with this approach is that the time delay required to relocate from the cycle ergometer to the seated dynamometer is often a minute or more, and considering that substantial recovery of muscle contractile function can occur within that time frame (Allen et al., 2010; Krüger et al., 2019; Sargeant et al., 1987), the magnitude of muscle fatigue at the task termination is likely to be underestimated. In addition, this relocation between ergometer and dynamometer limits the frequency at which fatigue assessments can be done. This often limits the measures to only pre‐ and post‐exercise, preventing any real insight into the time course of fatigue during the exercise bout itself. As the rate of muscular fatigue development during exercise is often of interest, some researchers have broken a single trial into multiple bouts performed on different days, with fatigue measurements at the end of each bout. In this way, the time course of muscle fatigue development can then be “pieced together” (Cannon et al., 2011; Gajanand et al., 2020; Keir et al., 2016). To circumvent these limitations, some members of our lab have developed and utilized a custom built semi‐recumbent cycle ergometer with locking pedals, removing the requirement to relocate to a seated dynamometer. This approach can allow muscle fatigue measurements within a few seconds of exercise cessation (Doyle‐Baker et al., 2018). This setup has allowed the measurement of muscular fatigue not only before and after an exercise task but also across the duration of the task as well, allowing the time course of fatigue development to be examined in much greater detail than done previously (Aboodarda et al., 2018; de Almeida Azevedo et al., 2022; Mira et al., 2018, 2020; Zhang et al., 2021). However, despite these improvements, there are some limitations with this approach. Firstly, it may be argued that the ecological validity of using a semi‐recumbent cycle ergometer is less than ideal when trying to generalize findings to real‐world cycling performance. Secondly, while this innovative setup has greatly improved our ability to monitor muscle contractile function during exercise, stopping the task every time a measurement must be made is disruptive, may disturb the underlying metabolic responses of interest, and may confound the development of fatigue from the task itself.

Therefore, the purpose of this study was to determine if muscle contractile function could be reliably quantified by the electrically evoked quadriceps contractions elicited during continued pedaling action during upright cycling. Additionally, we aimed to determine the validity of the method by comparing the kinetics of twitch force responses measured dynamically to those measured in isometric conditions. This method would allow muscle contractile function to be assessed during dynamic exercise nondisruptively, with zero time delay, and with better temporal resolution than has been done to date. We hypothesized that this method of monitoring muscle fatigue would agree well with isometric measurements performed at rest and would show good reproducibility between sessions.

## 2 MATERIALS AND METHODS

### 2.1 Participants

Eleven participants were recruited for this study. However, in two instances, the stimulating electrode was displaced prior to the completion of the trial, and so data for these participants have not been included in the current analysis. Therefore, data from nine participants (*n* = 7 males, *n* = 2 females, mean ± standard deviation: age 25 ± 6 years, height 175 ± 8 cm, and weight 74 ± 11 kg) were included in this study. All participants cleared the Physical Activity Readiness Questionnaire (PAR‐Q+) and were free of any health conditions or injuries that could prevent them from maximally exerting themselves. Additionally, participants were instructed to refrain from strenuous physical activity 24 h prior to testing and to avoid caffeine 12 h prior to testing. All participants provided written informed consent before commencing the study, which was approved by the institutional review board. The study was conducted in accordance with the Declaration of Helsinki (without registration).

### 2.2 Experimental protocol

**FIGURE 1:**
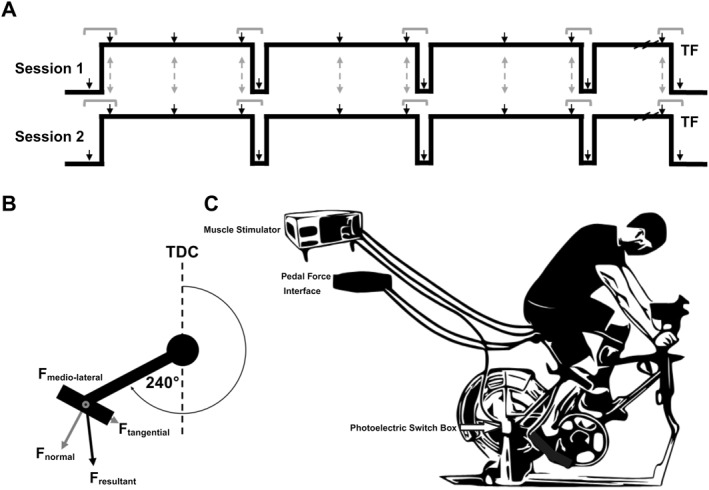
Illustration of experimental protocol. Panel (A) shows a schematic illustration of the experimental protocol. Downward black arrows indicate timepoints of stimulation. Isometric twitch measurements were taken prior to the trials, at ∼ 5 min intervals across the task, and at task failure, TF. Dynamic twitch measurements were taken at the onset of exercise, at the mid‐ and end‐points of each 5 min block, and prior to TF. Gray dashed arrows indicate time‐matched points for the dynamic twitch measurements across sessions, while gray brackets indicate time‐matched comparisons for the isometric versus dynamic twitch comparison. See text for more details. Panel B shows the diagram of pedal forces. Stimulation occurred at a crank angle of 240° (0° = top dead center. Twitch force amplitudes were measured from the resultant force (F_resultant_) as well its constituent components, the normal force (F_normal_), tangential force (F_tangential_) and medio‐lateral force (F_medio‐lateral_). Panel C illustrates the bicycle setup used during the experiment. TF, task failure.

Participants reported to the laboratory for three visits. The first visit was for a step incremental test to exhaustion to determine the lactate threshold, as well as to familiarize participants with the electrical stimulation procedures. Briefly, participants began cycling at a power output of 50 W, and power output was increased 25 W every 3 min until volitional exhaustion. Blood lactate concentration was measured via fingertip blood samples taken at the end of each stage (Lactate Plus, Nova Biomedical) and plotted against power output. The lactate threshold was estimated using the modified Dmax method (Bishop et al., 1998) in which the data point preceding a rise in blood lactate concentration ≥0.4 mmol/L, and all subsequent data points were fit with a third‐order polynomial curve, and the lactate threshold was identified as the point on the curve that yielded the maximum perpendicular distance from a straight line connecting the first and final data points (data not reported here). During this first visit, the seat and handlebar positioning was recorded for each participant and replicated for the subsequent trials. The following two visits were separated by at least 48 h, and both visits involved performing a constant‐load trial to task failure (TF) at a power output 10% above the estimated power output at lactate threshold. The mean ± standard deviation power output for these constant load trials was 202 ± 51 W, which represented 80.3 ± 6.6% of the highest power output achieved during the incremental test (range 70.3%–89.2%). These constant‐load trials began with a 3‐min warm‐up at 50 W, followed by a step increase to the prescribed power output. At 5‐min intervals throughout the trial, participants stopped pedaling for 15–30 s while a block was placed underneath the right pedal to obtain isometric twitch measurements at rest (see Figure 1A and “Assessment of muscle fatigue” below). For all testing sessions, participants were instructed to maintain a strict cadence of 80 rpm. All testing was performed on an electromagnetically braked cycle ergometer (Velotron RacerMate, SRAM LLC), outfitted with instrumented pedals able to measure force in three dimensions (Sensix), which was sampled at 500 Hz.

### 2.3 Assessment of muscle fatigue

**FIGURE 2:**
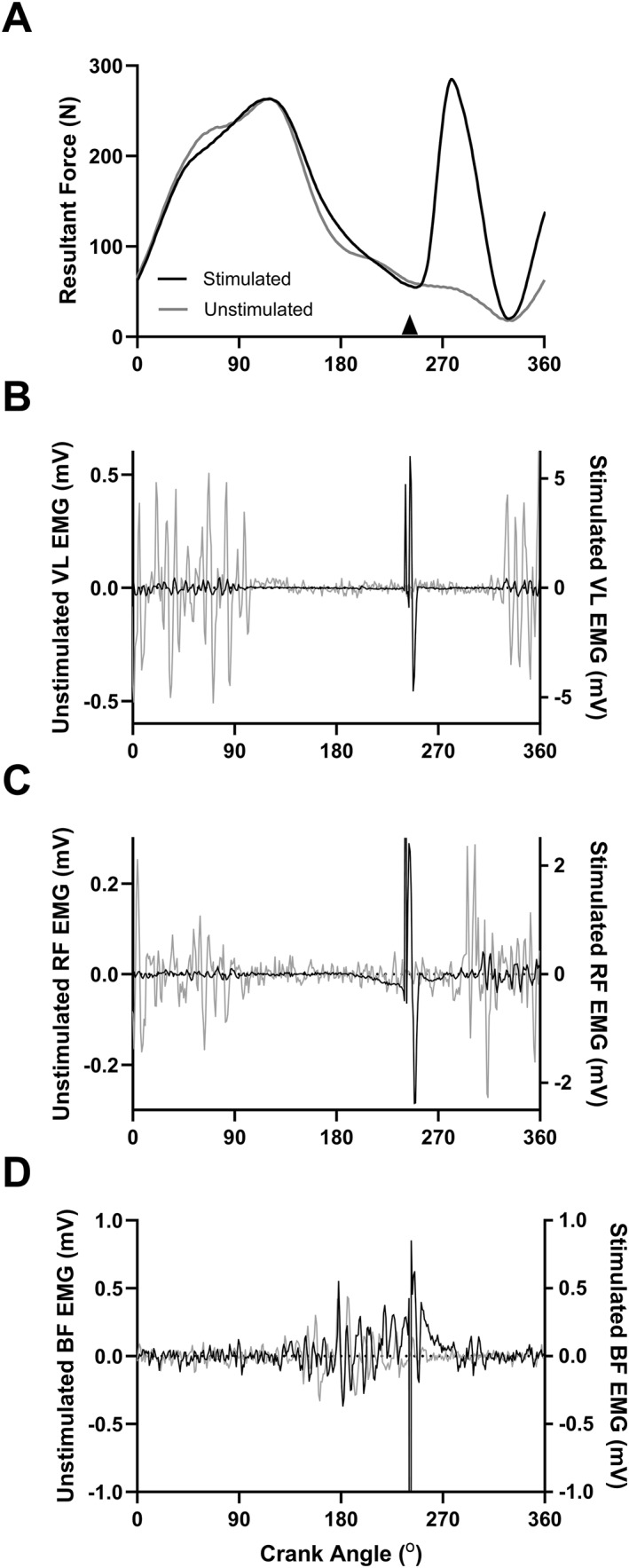
Representative example illustrating pedal force and muscle activation during stimulated and unstimulated pedal revolutions. Panel (A) shows example force traces across stimulated (black) and unstimulated (gray) pedal revolutions. The black arrow signifies the timing of stimulation. Panels (B, C, and D) show raw EMG traces for vastus lateralis, rectus femoris, and biceps femoris, respectively, from the same pedal revolutions as in panel A. To better illustrate the M‐wave and the voluntary muscle activity across the pedal strokes, stimulated and unstimulated revolutions are shown with different vertical axis scales.

To measure skeletal muscle fatigue, single square‐wave electrical stimuli (pulse duration: 1 ms; 400 V) were delivered to the femoral nerve percutaneously using a constant‐current stimulator (DS7A; Digitimer). The cathode (10 mm diameter; Kendall MediTrace; Covidien LLC) was placed on the right femoral triangle, and the anode (50 × 90 mm; Durastick Plus; DJO Global) was placed approximately halfway between the greater trochanter and the iliac crest. Following the placement of the stimulating electrode, a pad of gauze was pressed into the electrode and taped down with surgical tape. At the beginning of each session, with participants seated on the bike and a block placed underneath the right pedal at a crank angle of 90° (0° = top dead center (TDC)), current intensity was increased in 10–20 mA increments until a plateau in twitch force and vastus lateralis (VL) muscle compound action potential (M‐wave) amplitude was observed. The current intensity was then increased 30% to ensure complete motor unit activation during stimulation, and this intensity was used for the remainder of the session. The mean ± standard deviation stimulation current used for session 1 and session 2 were 110.5 ± 23.3 and 110.0 ± 19.6 mA, respectively. Isometric twitch force measurements were taken prior to the exercise bout as well as following each 5‐min cycling period. At each time point, the average of two twitches taken ∼5 s apart was used. To measure quadriceps twitch force during cycling, the cycle ergometer was outfitted with a custom‐made photoelectric switch mounted to the seat tube of the ergometer. On the inside of the chain ring, a piece of reflective paper triggered the switch at a crank angle of 240° (0° = TDC). The switch was connected to the electrical stimulator, as well as a PowerLab data acquisition device (Model 16/35, ADInstruments Inc, Colorado Springs, USA) in order to set the minimum time interval of 3 s between successive stimulations. An illustration showing the switch timing and ergometer setup is shown in Figure 1B,C. The crank angle of 240° was chosen for stimulation as EMG activity of the quadriceps has been shown to be near silent across this upstroke portion of the pedal cycle (da Silva et al., 2016; Dorel et al., 2008; Jorge et al., 1986), and because of several upstroke angles tested during pilot testing, this stimulation angle was determined to be the least disruptive to the pedal rhythm. Considering that voluntary quadriceps activation could alter the size of the twitch, independent of changes in contractile function (Merton, 1954), the absence of quadriceps activation at the time of stimulation was important for reliable twitch amplitude determination. Here, it may be noted that for the isometric twitch measurement, the choice to measure at a crank angle of 90° rather than approximate that of the dynamic measurement was due to the fact that during pilot testing, we found that when trying to block the pedals at a crank angle of 240°, it was less stable, and participants had a more difficult time fully relaxing their quadriceps prior to the stimulation. Additionally, the 90° angle more closely approximates the positioning utilized for typical knee extensor measurements on traditional seated dynamometers. A representative example illustrating the pedal force is shown in Figure 2A, and that of the electromyography (EMG) activity for the VL, rectus femoris (RF), and biceps femoris (BF) across the pedal stroke is shown in Figure 2B–D, respectively. Dynamic twitch force measurements were obtained at the very beginning of the bout, as soon as participants reached the proper cadence (between ∼10 and 20 s into the bout), at the mid‐ and endpoints of each 5‐min period (between ∼2:15–2:30 and ∼4:45–5:00 of each period, respectively), as well as prior to TF, following the participant signaling they were nearing completion. At each time point, three separate stimuli were given ∼3 s apart and the average of these measurements was used.

### 2.4 EMG

Self‐adhesive Ag/AgCl surface electrode pairs (10 mm diameter; Kendall MediTrace Covidien LLC), were placed over the muscle belly 20 mm apart (center to center) of the right VL and RF, just proximal to the myotendinous junction, and over the BF, approximately half the distance between the ischial tuberosity and the lateral epicondyle of the tibia. Electrodes were placed in the assumed orientation of the fibers, with a ground electrode placed over the patella. Prior to placing the electrodes, the skin was shaved, abraded, and cleansed with alcohol swabs to minimize resistance. EMG signals were recorded at a sampling rate of 2000 Hz using PowerLab (16/30‐ML880/P, ADInstruments), amplified, and bandpass filtered (5–500 Hz).

### 2.5 Data analysis

**FIGURE 3:**
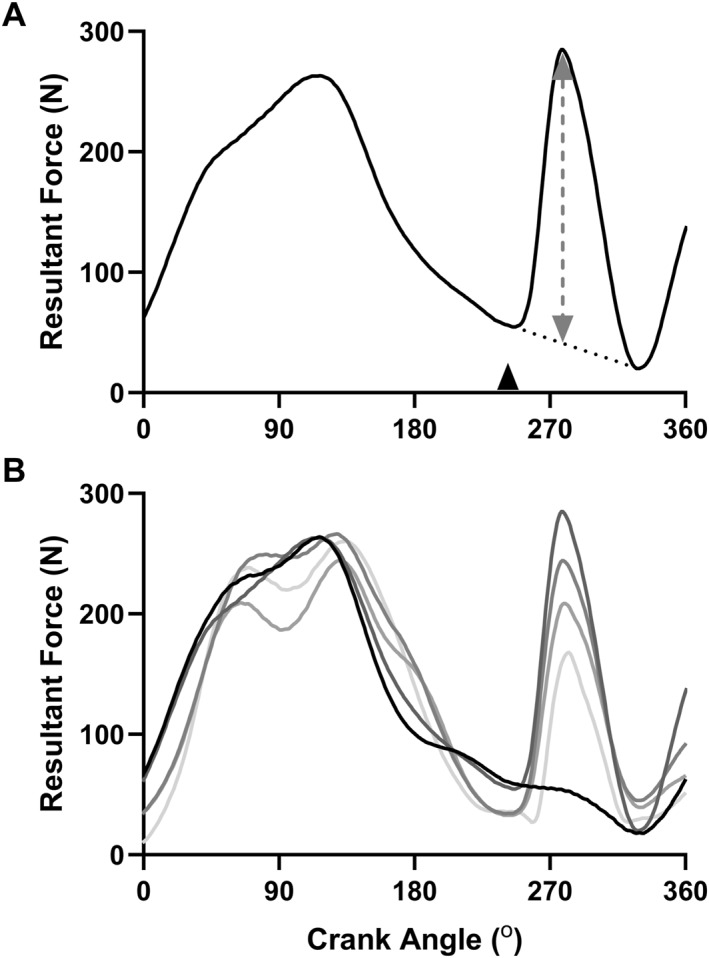
Measurement of dynamic twitch amplitudes during cycling. Panel (A) shows the calculation of twitch amplitude from a representative pedal stroke, whereby the baseline force was estimated from linearly interpolating the force between the onset and offset of the twitch, identified as the point at which the slope of the force trace equaled zero (dotted line). The black arrow signifies the timing of stimulation. Panel (B) shows several pedal force traces from the same participant measured at different timepoints across the fatiguing trial. Pedal force traces are from the right pedal, with 0° corresponding to top dead center. The black line shows an unstimulated pedal revolution, while the gray lines show those with evoked twitches shown sequentially from dark to light.

Data were collected in the I‐Crankset software (Sensix) and exported to LabChart (AD Instruments) for further analysis. For all twitch force measurements, twitch force amplitudes were measured from the resultant force (F_resultant_) as well its constituent components, the normal force (F_normal_), tangential force (F_tangential_) and medio‐lateral force (F_medio‐lateral_) (see Figure 1B). Isometric twitch amplitudes were calculated as the peak force minus the force preceding the stimulus. As the force applied to the pedals was changing around the period of stimulation, dynamic twitch amplitudes were calculated by subtracting a baseline force estimated from linearly interpolating the force between the onset and offset of the twitch, identified as the points at which the slope of the force trace was equal to zero, and solving for the time at which the peak twitch force occurred (see Figure 3A). Similar approaches to this have been utilized for calculating superimposed twitch amplitudes during shortening and lengthening contractions where voluntary force is changing across the stimulation period (Babault et al., 2001). For determining the test–retest reliability of the dynamic twitch amplitudes, for each participant, all data points up to the longest common timepoint between the two sessions for that individual were linearly interpolated between 0% and 100% of this time. For assessing the agreement between dynamic and isometric twitch amplitudes, only the resultant force was considered in the analysis. Here, isometric twitch measurements taken prior to the task, following each 5 min stage, and at TF, and the time‐matched dynamic twitch measurements were linearly interpolated between 0% (initial value) and 100% of TF (see Figure 1A for a schematic illustration of the stimulation timepoints and comparisons).

For the EMG data, maximal M‐wave amplitudes (M_max_) were determined for the VL and RF from the maximum peak‐to‐peak amplitudes for each stimulation during the pedaling action. As with the twitch amplitudes, for each timepoint, the average of three measures was used for the analysis and all data points up to the longest common timepoint between the two sessions for each individual were linearly interpolated between 0% and 100% of this time. M‐waves measured during the isometric measurements were not considered in the analysis.

### 2.6 Statistical analysis

Data are presented as mean ± standard deviation. Statistical analyses were conducted using R v 4.3.0 and IBM SPSS, v 27 (IBM Corp.). Shapiro–Wilk tests were performed to assess data normality for the dependent variables. A robust linear mixed effects model using the *robustlmm* package v 3.2.3 in R was used to assess any differences in the dynamic twitch amplitudes and M‐wave amplitudes across days. Here, two conditions (session 1 and session 2) and 12 timepoints (10% increments between 0 (i.e., initial measurement) and 100% of the common trial length as well as at TF) were compared, with participants added as a random effect. Similarly, for comparing the isometric versus dynamic F_resultant_ twitch responses, a robust linear mixed‐effects model was run between two conditions (isometric and dynamic) and 11 timepoints (10% increments between 0% and 100% of the total trial duration). For this analysis, all complete trials from both sessions were pooled together and participants were included as a random effect to account for the within‐subject dependencies across the duplicate trials. Relative reliability was assessed using the intraclass correlation coefficient (ICC) (two‐way mixed effects, single measurement, and absolute agreement) and absolute reliability was assessed by the within‐subject coefficient of variation (CV), using the root mean square approach (CV_RMS_) (Bland, 2006). According to Bland (2006), calculating the within‐subject CV simply as the overall average CV estimated from each participant (CV_AVG_) provides an underestimation of the true within‐subject variation. However, this measure was included here so as to provide a more direct comparison with similar studies where this approach was used. For the test–retest reliability of the dynamic twitch force, the dependent variables were the twitch force at the initial time point as well as the twitch force at the longest common time point and that at TF, both of which were compared as an absolute value and as a percentage of the initial value. Similarly, for the agreement between isometric and dynamic conditions, the dependent variables were the twitch force at the initial timepoint, as well as the twitch force at TF, again expressed as both an absolute value and as a percentage of the initial value. Here, it is worth noting that significant differences were observed in the extent of decline in twitch force between dynamic and isometric conditions. It was considered that this effect may be due to differing degrees of potentiation for the initial measurement, and so an additional analysis was performed to assess the agreement between conditions when the value at TF was expressed as a percentage of the value at the 5 min timepoint (i.e., when potentiation would be similar between conditions) (see results and discussion for more details). Inter‐day reliability for the VL and RF M_max_ was assessed from the value for the initial timepoint for session 1 versus session 2. ICC values were interpreted as poor (<0.5), moderate (0.5–0.75), good (0.75–0.90), and excellent (>0.90) (Koo et al., 2016). For the test–retest reliability of time to TF and dynamic twitch forces, systematic bias between sessions was tested using paired *t* tests, while a mixed model was used to detect differences between dynamic and isometric conditions to account for the within‐subject dependencies. Significance was set at *α* = 0.05.

## 3 RESULTS

**FIGURE 4:**
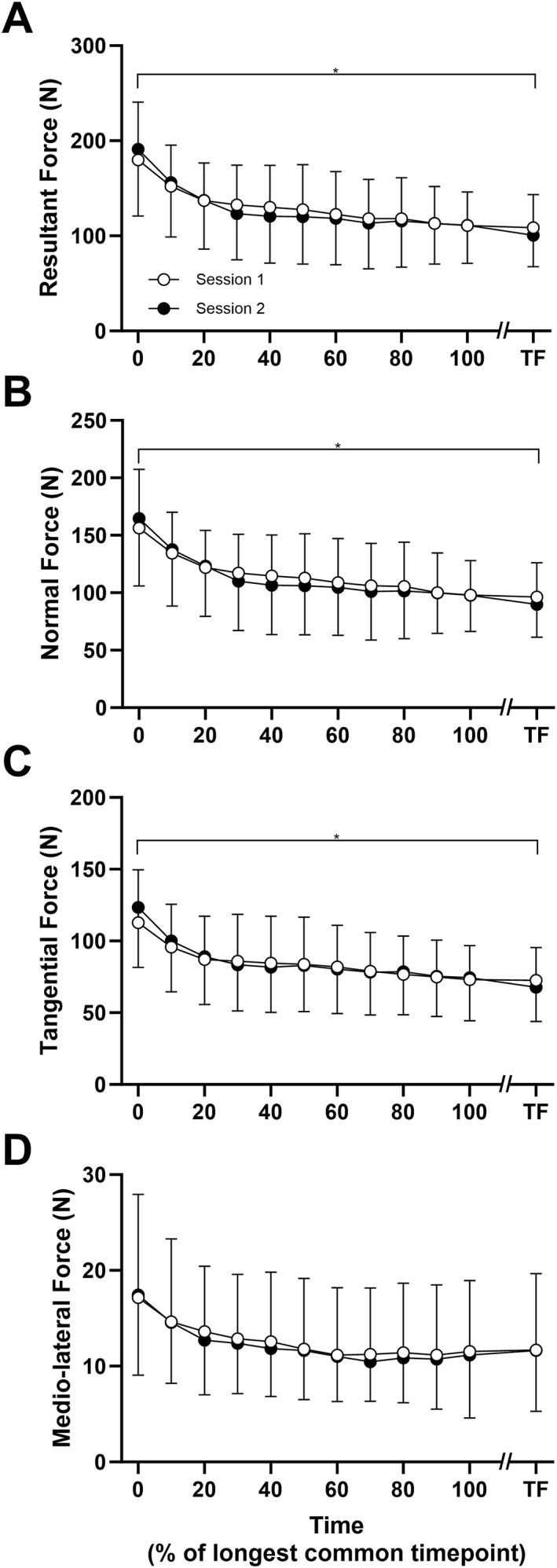
Repeatability of dynamic twitch forces across a fatiguing cycling trial. The group mean (±SD) dynamic twitch forces across session 1 and session 2 are shown in the figure. Data for *n* = 9 participants were interpolated between 0% and 100% of the longest common timepoint between session 1 and session 2. TF, task failure.

There was no difference in time to TF between session 1 (21.3 ± 8.9 min) and session 2 (24.2 ± 13.8 min) (*P* = 0.329). Across both sessions, the time to TF ranged from ∼10 to ∼55 min, corresponding to an average of 9.7 ± 4.2 dynamic measurements, and 5.6 ± 2.2 isometric measurements. For F_normal_, F_tangential_, and F_resultant_, there was a significant main effect of time (*P* ≤ 0.002) but no session (*P* ≥ 0.435) or interaction effect (*P* ≥ 0.279). F_medio‐lateral_ showed no time effect (*P* = 0.109), session effect (*P* = 0.995), or interaction (*P* = 0.706). The group mean dynamic twitch forces for each of the components F_resultant_, F_normal_, F_tangential_, and F_medio‐lateral_, interpolated between the initial value and the each participant's longest common time point between session 1 and session 2 as well as that at TF are shown in Figures 4A–D, respectively. Table 1 shows the test–retest reliability measures for the dynamic twitch forces between session 1 and session 2.

**TABLE 1:** Test–retest reliability of the resultant dynamic twitch force and its three‐dimensional components measured during two fatiguing constant‐load cycling tasks.

Variable	Session 1	Session 2	CV_RMS_ (%) (95% CI)	CV_AVG_ (%) (95% CI)	ICC (95% CI)
Resultant force					
Initial force (*N*)	180.1 ± 60.5	191.0 ± 70.0	9.9 (5.8–14.1)	7.5 (2.9–12.1)	0.920 (0.705–0.981)
Force at final common timepoint					
(*N*)	110.6 ± 32.9	109.3 ± 36.4	14.5 (8.2–18.8)	12.7 (7.7–17.7)	0.842 (0.433–0.962)
(% of initial)	64.7 ± 21.2	61.5 ± 20.7	17.6 (7.8–23.6)	14.7 (7.8–21.6)	0.705 (0.127–0.925)
Force at task failure					
(*N*)	108.6 ± 34.9	100.6 ± 32.9	13.4 (6.7–18.3)	11.5 (6.1–16.9)	0.846 (0.491–0.962)
(% of initial)	62.5 ± 17.6	55.9 ± 18.5	18.1 (7.9–24.4)	15.1 (8.0–22.2)	0.678 (0.139–0.915)
Normal force					
Initial force (*N*)	156.4 ± 51.0	164.7 ± 58.8	9.9 (2.7–13.8)	7.7 (3.2–12.2)	0.916 (0.696–0.980)
Force at final common timepoint					
(*N*)	98.1 ± 29.9	97.7 ± 31.3	11.8 (5.6–15.7)	10.0 (5.7–14.4)	0.870 (0.517–0.969)
(% of initial)	65.5 ± 19.8	62.6 ± 19.2	15.0 (3.7–20.9)	12.2 (5.9–18.4)	0.723 (0.165–0.930)
Force at task failure					
(*N*)	96.5 ± 29.7	90.0 ± 28.7	12.6 (7.2–16.3)	10.9 (6.4–15.4)	0.842 (0.482–0.961)
(% of initial)	63.5 ± 16.0	57.6 ± 18.5	16.7 (8.0–22.5)	14.1 (7.6–20.7)	0.669 (0.120–0.912)
Tangential force					
Initial force (*N*)	113.1 ± 36.3	123.4 ± 41.9	9.6 (1.7–13.4)	7.7 (3.6–11.8)	0.906 (0.578–0.979)
Force at final common timepoint					
(*N*)	73.1 ± 23.5	74.4 ± 30.1	15.1 (8.8–19.5)	13.0 (7.5–18.5)	0.855 (0.476–0.965)
(% of initial)	67.1 ± 20.3	61.4 ± 19.2	18.7 (2.9–26.2)	14.9 (6.8–22.9)	0.669 (0.108–0.913)
Force at task failure					
(*N*)	72.6 ± 22.8	67.8 ± 23.8	11.2 (6.4–14.5)	9.8 (6.0–13.6)	0.916 (0.670–0.980)
(% of initial)	66.5 ± 18.6	56.2 ± 15.0^a^	17.1 (5.9–23.5)	13.0 (5.1–20.9)	0.656 (0.016–0.912)
Medio‐lateral force					
Initial force (*N*)	17.2 ± 10.8	17.4 ± 8.4	20.1 (0.4–28.4)	15.4 (6.2–24.6)	0.943 (0.768–0.987)
Force at final common timepoint					
(*N*)	11.6 ± 7.4	11.2 ± 6.6	30.9 (20.1–38.9)	28.0 (18.7–27.4)	0.734 (0.163–0.934)
(% of initial)	77.9 ± 37.7	69.3 ± 34.9	27.9 (16.0–36.0)	24.7 (15.5–33.9)	0.787 (0.347–0.947)
Force at task failure					
(*N*)	11.7 ± 8.0	11.6 ± 6.4	30.0 (18.8–38.0)	26.6 (16.9–36.4)	0.737 (0.165–0.935)
(% of initial)	75.5 ± 34.9	74.5 ± 36.2	19.1 (11.6–27.5)	14.5 (5.6–23.4)	0.908 (0.645–0.979)

*Note*: Data are expressed as ± SD (*n* = 18 trials).

Abbreviations: CI, confidence interval; CV_AVG_, coefficient of variation calculated as the average from each participant; CV_RMS_, coefficient of variation calculated using the root mean square approach; ICC, intraclass correlation coefficient.

^a^ Indicates a significant difference between trial 1 and trial 2.

**FIGURE 5:**
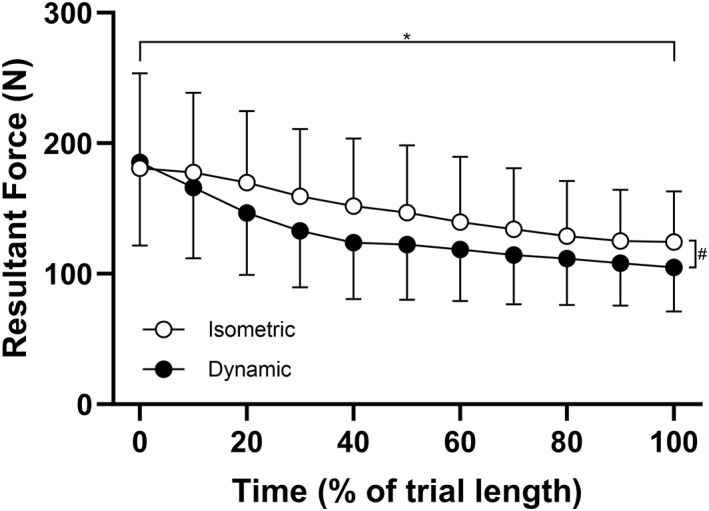
Agreement between the resultant dynamic and isometric twitch forces across a fatiguing cycling trial. The group mean (±SD) twitch forces measured isometrically and while pedaling are shown in the figure. Data from *n* = 9 participants were interpolated between 0% and 100% of the time to task failure. *indicates significant main effect of time. TF, task failure. ^#^ indicates significant main effect of condition (dynamic vs. isometric).

In comparing the dynamic to isometric twitch forces, there was a significant main effect of time (*P* < 0.001) and condition (*P* < 0.001) but no interaction effect (*P* = 0.740) (Figure 5). The agreement between the isometric and dynamic twitch forces are shown in Table 2. There were no systematic differences between the initial isometric or dynamic twitch amplitudes (*P* = 0.601); however, at TF, dynamic twitch forces had declined to a greater extent than isometric twitch forces, both when expressed as absolute values (*P* = 0.002) and as a percentage of the initial value (*P* = 0.001). The discrepancy between the dynamic and isometric conditions was an unexpected result; however, it was considered that this may be explained by differences in the degree of potentiation for the initial timepoint. While the isometric condition was first measured following a period of passive rest, the dynamic condition was measured after ∼10–20 s of cycling at a relatively high power output. Therefore, in exploring the agreement between the extent of decline at TF between the dynamic and isometric conditions, it was decided to perform an additional analysis with the value at TF expressed relative to the measurement at the 5‐min timepoint, when the level of potentiation would be expected to be similar. This comparison revealed no difference between conditions (*P* = 0.207).

**TABLE 2:** Agreement between isometric and dynamic quadriceps resultant twitch amplitude measures during a fatiguing constant‐load cycling task.

Variable	Isometric	Dynamic	CV_RMS_ (%) (95% CI)	CV_AVG_ (%) (95% CI)	ICC (95% CI)
Initial twitch force (*N*)	180.8 ± 72.8	185.5 ± 63.8	15.8 (9.2–20.3)	12.5 (5.8–19.1)	0.833 (0.609–0.934)
Twitch force at task failure					
(*N*)	124.4 ± 38.6^a^	104.8 ± 33.6	20.7 (12.8–26.3)	16.6 (8.1–25.1)	0.644 (0.169–0.861)
(% of initial)	73.6 ± 23.5^a^	59.3 ± 18.1	19.8 (12.6–25.1)	16.0 (7.9–24.1)	0.607 (0.031–0.854)
(% of value at 5 min)	79.8 ± 11.8	86.6 ± 17.7	13.3 (2.8–18.5)	8.7 (2.8–18.5)	0.357 (−0.072–0.690)

*Note*: Data are expressed as ± SD (*n* = 18 trials).

Abbreviations: CI, confidence interval; CV_AVG_, coefficient of variation calculated as the average from each participant; CV_RMS_, coefficient of variation calculated using the root mean square approach; ICC, intraclass correlation coefficient.

^a^ indicates a significant difference between isometric and dynamic conditions.

**FIGURE 6:**
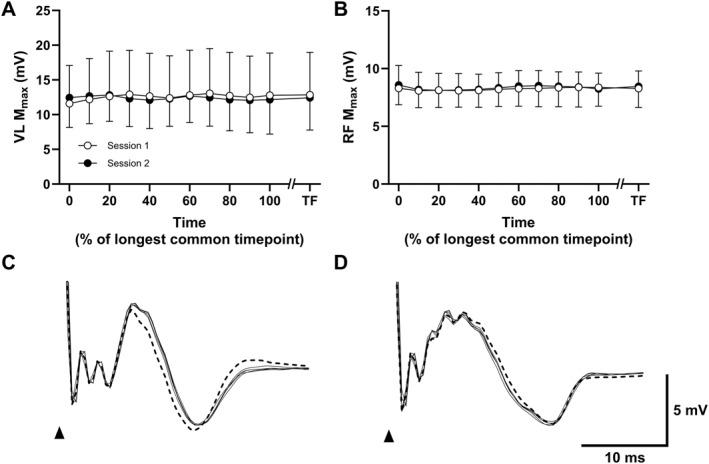
Repeatability of maximal M‐wave amplitudes (M_max_) for the vastus lateralis (VL) and rectus femoris (RF) across a fatiguing cycling trial. Panels A and B show the group mean (±SD) maximal M‐wave amplitudes for VL and RF, respectively, across session 1 and session 2. Data for *n* = 9 participants were interpolated between 0% and 100% of the longest common timepoint between session 1 and session 2. Panels C and D show superimposed M‐wave traces for the VL and RF, respectively, from a representative participant for each measured timepoint across a task. In this example, the participant reached task failure in ∼13 min, corresponding to 6 stimulation timepoints. The thicker dashed trace is that measured at the onset of exercise, while the remaining timepoints are shown as thin black traces. The black arrow signifies the timing of stimulation. TF, task failure.

For the EMG variables, both VL and RF M_max_ showed no significant time effect (*P* ≥ 0.389), session effect (*P* ≥ 0.428), or interaction (*P* ≥ 0.494). Inter‐day reliability for VL M_max_ was poor (CV_AVG_ = 27.0% and ICC = 0.232), while that for RF, M_max_ was moderate (CV_AVG_ = 8.2% and ICC = 0.601). The group mean VL and RF M_max_ across the trials are shown in Figure 6A,B, respectively, and the changes in the VL and RF M‐wave shape across a trial for a representative participant is shown in Figure 6C,D, respectively.

## 4 DISCUSSION

The purpose of this study was to determine the validity and reliability of electrically evoked quadriceps twitch force elicited during a fatiguing cycling task. Overall, the dynamic twitch amplitudes showed good to excellent relative reliability, with ICCs typically >0.75 and CVs typically <15%. Interestingly, there were some discordant results between twitch responses measured while cycling with those measured isometrically at rest; however, much of the disagreement was removed when the data were normalized to the 5‐min timepoint, where we presumed the degree of potentiation between the two conditions would be more similar. These results demonstrate that this method can provide a reliable means to monitor muscle contractile function in real time in a nondisruptive manner.

Several studies utilizing seated isometric dynamometers to measure knee extension force have reported the inter‐day reliability for quadriceps twitch force in an unfatigued state, with CVs ranging from 4.3% to 12.6% and ICCs ranging from 0.80 to 0.96 (Amann et al., 2008; Bachasson, Millet, et al., 2013; Doyle‐Baker et al., 2018; Kufel et al., 2002; Nyberg et al., 2018; Place et al., 2007; Thomas et al., 2016; Tofari et al., 2016; Varesco et al., 2021). In the present study, the resultant force for the unfatigued dynamic twitches evoked at baseline measurement had an average CV of 7.5% and ICC of 0.920, showing that the reliability of this method is commensurate with what has become the standard means of assessing muscle contractile function before and after cycling exercise. Comparatively, fewer studies have looked at the test–retest reliability of quadriceps twitch force following a fatiguing task, which introduces the potential for more variability in the measurement. On the lower end, (Bachasson, Millet, et al., 2013) reported a CV of 5.1% following an exhaustive isometric leg extension test, while Place et al. (2007) reported a CV of 9.3% in the potentiated quadriceps twitch force following a 2 min maximal isometric voluntary contraction (MVC). Comparitively, Doyle‐Baker et al. (2018) reported a CV of 14.2% immediately following an incremental cycling test, while Varesco et al. (2021) reported a CV of 16.6% following a fatiguing dynamic leg extension task. In comparison, the CV for the resultant force determined at TF in the present study was 11.5%. In looking at these results, it would appear that tests utilizing dynamic measurements compared to purely isometric contractions have the potential to introduce more variability across the duration of the task. One potential explanation for this is the likelihood of subtle electrode movement relative to the femoral nerve due to limb movement during the task, and indeed, we had two instances where the stimulating electrode was displaced partway through the trial, preventing us from analyzing these data. While we attempted to sufficiently secure the stimulating electrode with gauze and surgical tape to prevent any movement during the trials, a stronger adhesive perhaps would have improved the consistency in our results and should be considered when using this method in the future. Apart from these concerns, another challenge with stimulation during cycling is that the baseline force is variable, which may have the potential to introduce more measurement variability than those obtained on a traditional seated dynamometer. An additional consideration is that due to it being measured during a dynamic task, co‐contractions of antagonist muscles during the stimulation may impact the resultant twitch force. For example, hamstring muscle activation during the upstroke portion of the pedal cycle may counteract knee extension torque, and thus any variability in knee flexion torque could concomitantly increase the variability in the observed twitch forces. However, contrary to these concerns, our results show that both in a fresh and fatigued state, our method provides comparable results to the gold standard and with the obvious advantages of it being nondisruptive to the task and more ecologically valid for cycling exercise.

When comparing the dynamic twitch forces to those measured isometrically, the relative agreement between measures was generally moderate to good; however, we did observe a significantly greater decline in the dynamic twitch force across the task. The variability in these two measures can likely be explained by three primary factors. Firstly, some of the within‐subject variations between the two measures may be because the muscle was lengthening while being stimulated in the dynamic condition, which could be expected to alter the twitch amplitude compared to in an isometric state (Behrens et al., 2016). A second consideration is that the two conditions were assessed at different crank angles; dynamic twitches were stimulated at 240°, while isometric twitches were stimulated at 90°. As mentioned earlier, a primary factor in the decision to use a crank angle of 240° for the dynamic twitches was the minimization of quadriceps activation during this portion of the pedal stroke. However, for the isometric twitch measurement, a crank angle of 90° was chosen to try to approximate the positioning utilized for typical knee extensor measurements on traditional seated dynamometers, as well as the fact that it was found to be easier to fully relax the quadriceps at this angle compared to greater crank angles tested in pilot measurements. Therefore, some of the individual variation between the measures may be explained by this difference in crank angle, which may subsequently impact joint angles and quadriceps muscle lengths.

As alluded to in the results, a final consideration which may explain some of the general variability between dynamic and isometric conditions as well as specifically the greater relative decline in twitch force in the dynamic condition is differences in the magnitude of potentiation between conditions. Activity‐dependent potentiation refers to an enhanced contractile response which results from prior contractile activity (Macintosh et al., 2002) and is typically ascribed to an increase in Ca^2+^ sensitivity due to phosphorylation of the myosin regulatory light chains (Houston et al., 1985). Importantly, potentiation can coexist with fatigue (MacDougall et al., 2020; Rassier et al., 2000), and moreover, potentiation is stimulation frequency‐dependent, whereby the relative enhancement in the contractile response is greatest at lower stimulation frequencies (e.g., single stimuli, as used in this study) and gradually reduces as the stimulation frequency increases (MacDougall et al., 2020; MacIntosh et al., 2000). In the present study, the initial measurement for the isometric condition was performed following a period of rest, while that for the dynamic condition was performed following ∼10–20 s of cycling, presumably leading to greater levels of potentiation than the prior isometric measure. In this regard, Kufel et al. (2002) directly compared potentiated twitches following a 5 s MVC to unpotentiated twitches in the detection of quadriceps muscle fatigue across a fatiguing task and found that the potentiated twitch exhibited an earlier and significantly greater reduction in twitch force across the task, concluding that the potentiated twitch is superior for detecting early muscle fatigue. Interestingly, Kufel et al. (2002) reported that the average percent decline in twitch force was 12.4% greater for the potentiated twitch than the unpotentiated twitch, which is quite similar to our results showing that when twitch force was expressed as a percentage of the initial value, there was on average a 14.3% greater reduction in the dynamic condition at task failure. It may be noted that in most experimental setups utilizing a traditional seated dynamometer, twitch measurements are performed following an MVC as part of a more involved assessment of neuromuscular fatigue (e.g., see Ref Place et al., 2007), and thus begin from a maximally potentiated state. However, (while noting it was necessary to include isometric measurements as a point of comparison within the current study), the ultimate aim of our setup is to be able to assess muscle contractile function while cycling continues entirely uninterrupted, thus precluding the use of intermittent MVCs across the bout. Thus, any potentiation effects utilizing the current setup are unlikely to be maximized and could conceivably vary over time due to factors such as altered motor unit recruitment. Nonetheless, the similarities between our results and those of Kufel et al. (2002) suggest that the present method provides a measure of muscle fatigue with a similar sensitivity to that performed from a fully potentiated state, at least while cycling at the intensities utilized in this study. In addition to potentiation effects, another consideration which may be expected to lead to differences in the relative decline in force between the isometric and dynamic conditions is that some recovery may have occurred between stopping pedaling and the isometric twitch force measurement. Although across all trials, this time interval averaged only 4.3 ± 1.5 s, it has been shown that substantial recovery may take place even in the initial seconds following exercise (Allen et al., 2010; Sargeant et al., 1987; Westerblad et al., 1991), and so, this may have also been a factor in the relatively reduced decrement in force we observed in the isometric condition. As an aside, this point also highlights the advantages of eliciting stimuli during cycling over others where exercise stoppage is unavoidable.

Despite the advantages of measuring muscle contractile function in dynamic conditions, with high temporal resolution and in a nondisruptive manner, it is not without limitations. Perhaps, the greatest limitation is that by solely employing electrically evoked contractions to measure fatigue, only peripheral fatigue (i.e., modulation at or below the neuromuscular junction) can be assessed, precluding any measurement of maximal voluntary force as well as limiting insight into central factors (i.e., alterations in central motor output) that may be simultaneously occurring and acting as contributing factors in limiting exercise tolerance (Iannetta et al., 2022). With this in mind, considering that central fatigue plays a comparably smaller role than peripheral fatigue in limiting exercise tolerance at high intensities (Burnley et al., 2018; Iannetta et al., 2022; Thomas et al., 2016), together with the fact that the existence of ongoing potentiation may mask some of the muscle fatigue accrued, it may be suggested that this method would be best suited for use during high intensity cycling exercise, where relatively large magnitudes of peripheral fatigue would be expected and would likely be the primary limiter of endurance. On the other hand, utilizing a standardized means of potentiating the muscle, such as including a brief period of higher intensity cycling prior to the bout (and potentially intermittently during the bout as well), may conceivably permit this method to be used at lower intensities as well.

An additional critique to this method could potentially be the use of single stimuli for assessing muscle contractile function. Indeed, some have suggested that when assessing twitch responses, doublet stimulation should be used instead of single twitches, due to them being less susceptible to potentiation effects (Hansen et al., 2003), and less variable than single twitch responses when superimposed onto voluntary contractions (Suter et al., 2001) (although, single twitch responses are still very often the sole means of assessing muscle contractile function (e.g., Refs Amann, 2011; Amann et al., 2008; Amann et al., 2009; Amann et al., 2013; Thomas et al., 2014; Thomas et al., 2016). On the other hand, when measured at rest, variability in single or double stimuli has shown to be similar (Place et al., 2007). In our setup, when stimulating at a crank angle of 240°, voluntary quadriceps activation is negligible (Dorel et al., 2008; da Silva et al., 2016; Jorge et al., 1986), and so any effects of prior voluntary activation per se on twitch amplitude variability are likely to be minimal. Aside from these considerations, a practical concern with this method was the tolerability of multiple stimulations across the cycling bout as well as the potential disruption to the pedaling rhythm that may occur with higher force responses, which was one factor in the decision to use single twitches. These considerations aside, it is conceivable that both potentiation effects (Boullosa et al., 2018; Inglis et al., 2011) as well as low‐frequency fatigue (Efthimiou et al., 1986; Stokes et al., 1989) may impact endurance performance. Importantly, a single twitch measure would be more sensitive to changes in both of these factors (and their coexistence [Fowles et al., 2003; Rassier et al., 2000]) than an evoked response from a higher frequency stimulus and/or in a fully potentiated state would. Therefore, it may be argued that a single‐twitch measure performed in a “natural” state of potentiation, as done here, could provide a more accurate reflection of the current functional status of the muscle. Nevertheless, it may still be of interest to explore the impact of altering the stimulation parameters used in this study to determine what other information may be gleaned.

## 5 CONCLUSION

In conclusion, here, we have reported a novel method of measuring muscle fatigue in dynamic conditions with comparable reliability to measures taken on traditional seated dynamometers often utilized to measure muscle fatigue following cycling exercise. Importantly, however, this method offers clear advantages over the current gold standard procedure, as it allows exercise to go on uninterrupted while muscle fatigue is being measured in real time, providing an intriguing new approach to explore the kinetics of muscle fatigue during cycling exercise.

## CONFLICT OF INTEREST STATEMENT

The authors declare that they have no conflicts of interest.

## Data Availability

The datasets generated during and/or analyzed in the current study are available on request from the corresponding author.
